# Supplementary material for: Mortality and its associated factors in transfused patients at a tertiary hospital in Uganda
Source: PLoS One. 2022 Sep 22;17(9):e0275126. doi: 10.1371/journal.pone.0275126 (PMC9499229; doi:10.1371/journal.pone.0275126)
Supplement: Attachment — Submitted filename: Reviewers comments.docx [file pone.0275126.s002.docx]

Thank you for affording me this opportunity to review this manuscript. Kindly see below comments and queries for your attention.

| **Line No.** | **Author’s statement** | **Reviewer’s comment** |
| --- | --- | --- |
| 70 | *“…such as transmission of* ***diseases*** *like hepatitis B and C…”* | Please use the word “**infections**” rather than “**diseases**” |
| 70-71 | *“…human 71 immunodeficiency virus.”* | Please add the abbreviation “HIV” in brackets and use it henceforth. |
| 71 | *“…bacteria [2] and malaria [3] as well as…”* | Please add a comma after malaria. |
| 77 | *“…limited blood supply is a major challenge”* | Is this challenge limited to Sub-Saharan Africa? Or a particular country? Or a continent? Or low-to-middle income countries? |
| 79 | *“…most blood transfusion in these settings are with blood that is less than 14 days”* | Is this a good or a bad thing? |
| 80 | *“A randomized, controlled trial conducted in six large hospitals in four countries…”* | Countries from where? SSA? Africa? Europe? A particular region? Etc. |
| 81-82 | *“…increased mortality in patients transfused with fresh red blood cells compared to older red blood cells”* | What is “fresh” red blood cells?  What is “old” red blood cells? |
| 88-89 | *“We, therefore, undertook a study to describe the mortality and its associated factors in patients transfused with* ***whole blood****…”* | Please confirm that all your study participants received whole blood and not red cell concentrates or other individual blood products. |
| 93-94 | *“…study conducted at the Uganda Cancer Institute (UCI) and the internal medicine unit of…”* | It looks like most of your study participants were immunocompromised (HIV and/or malignancy). I would suggest you add a line or so in the background section on the risks of transfusing whole blood in such population. |
| 99-100 | *“…tumours are* ***treated*** *at the UCI; whereas patients with general internal medicine conditions are* ***taken care of*** *in MNRH* | I would suggest you use the word “managed” for both “treated” and “taken care of” for uniformity. |
| 100-101 | *“…generally follow the same guidelines from the Ugandan ministry of health which recommends…”* | Please add a comma after the word “health”. |
| 103 | *“…haemoglobin level is <7 g/dL or 6 for patients with…”* | 6 what? Please add the units. |
| 104 | *“Blood components for the two hospitals are* ***prepared*** *and provided…”* | Please use the word “processed” instead of “prepared”. |
| 105-107 | *“Prior to delivery to the hospitals, all blood is serologically tested to be negative for human immunodeficiency virus, hepatitis B and C, and syphilis.”* | Please rephrase:  i.e., "Every donated blood products/ unit is tested for HIV, hepatitis B, hepatitis C, and syphilis, and can only be issued to the hospital once all the tested serological markers are negative".  Please ensure that no nucleic acid testing is performed on these units before committing yourself to only serological testing. |
| 110-111 | *“…immediate spin cross-match – all performed using the tile method which is standard of care in SSA settings”* | Which one is the standard of care? The time method or just the cross-match? |
| 131 | *“…median and IQR for nonparametric data”* | Please spell IQR in full the first time. |
| 144-145 | *“Baseline haemoglobin levels, stages of cancers and HIV characteristics were not included since they were not consistently stated…”* | Baseline haemoglobin could have been a very valuable piece of information in this study. I’m not sure how readily available this information can be from the blood bank? |
| 151-152 | *“The top five common diagnoses were isolated HIV related anaemia…”* | Parvovirus B19 infection is very common in this group of patients, and requires treatment with IVI immunoglobulins rather than blood transfusion. Is there any information in this regard? |
| 153 | *“…unexplained anaemias, 186 (9.2)…”* | Please add the units for 9.2 |
| 154 | *“Most patients received 1 unit of blood…”* | Write “1” in words.  Write “whole blood” if it is whole blood. |
| 209 | *“In this retrospective study of patients transfused with whole blood in Uganda…”* | Again, please confirm that all your study participants received whole blood and not red cell concentrates or other individual blood products. |
| 227 | *“A systematic study to assess the association of* ***red blood cell transfusion and*** *in-hospital mortality…”* | You probably might want to also look at the literature about “whole blood” transfusion. |
| 231 | *“(red blood cells, RBCs)”* | Please omit “red blood cells” |
| 250 | *“…all patients in our study received non-leucoreduced whole blood.”* | Please add a line or so in the background about the risks of transfusing “non-leucoreduced whole blood.” |
| 260-262 | *“…the underlying disease condition in our study may simply reflect the inherent influence of the specific disease entity on mortality,* ***rather than the effect of blood transfusion.”*** | I would suggest that the authors add in the background:   - The risks of blood transfusion. - Advantages and disadvantages of transfusing whole blood vs RBC (leuco-depleted vs unfiltered). - Clinical impact of anaemia in immunocomromised patients   Please add in the discussion whether your hospitals do have any “haemovigilance” committee, or any designated person who oversees the transfusion-associated reactions. |
| 278-279 | *“…patients who received additional blood had reduced mortality rates.”* | Is it possible that “inadequate” transfusion might have been a contributing factor in the higher mortality amongst those participants who received only one unit of whole blood? |
