# Supplementary material for: Mortality and its associated factors in transfused patients at a tertiary hospital in Uganda
Source: PLoS One. 2022 Sep 22;17(9):e0275126. doi: 10.1371/journal.pone.0275126 (PMC9499229; doi:10.1371/journal.pone.0275126)
Supplement: Attachment — Submitted filename: Response to Reviewers.docx [file pone.0275126.s003.docx]

**RESPONSE TO REVIEWERS COMMENTS**

Dear Reviewer(s),

Thank you for the thorough review of our manuscript. We have addressed your concerns as follows:

**Reviewer 1**

Your comments have been left intact, our responses are in the last column with dark highlight.

| **Line No.** | **Author’s statement** | **Reviewer’s comment** | **Authors’ Reponses** |
| --- | --- | --- | --- |
| 70 | *“…such as transmission of* ***diseases*** *like hepatitis B and C…”* | Please use the word “**infections**” rather than “**diseases**” | Addressed and revised whole paragraph |
| 70-71 | *“…human 71 immunodeficiency virus.”* | Please add the abbreviation “HIV” in brackets and use it henceforth. | Addressed and  updated subsequent HIV |
| 71 | *“…bacteria [2] and malaria [3] as well as…”* | Please add a comma after malaria. | Addressed |
| 77 | *“…limited blood supply is a major challenge”* | Is this challenge limited to Sub-Saharan Africa? Or a particular country? Or a continent? Or low-to-middle income countries? | Specified SSA in line 81 |
| 79 | *“…most blood transfusion in these settings are with blood that is less than 14 days”* | Is this a good or a bad thing? | Addressed in the sentence beginning from line 84 “A randomized, controlled trial conducted in six hospitals in North America, Australia and Israel…” |
| 80 | *“A randomized, controlled trial conducted in six large hospitals in four countries…”* | Countries from where? SSA? Africa? Europe? A particular region? Etc. | Added North America, Australia and Israel |
| 81-82 | *“…increased mortality in patients transfused with fresh red blood cells compared to older red blood cells”* | What is “fresh” red blood cells?  What is “old” red blood cells? | Revised to red blood cells stored for a shorter time vs those store for a longer time |
| 88-89 | *“We, therefore, undertook a study to describe the mortality and its associated factors in patients transfused with* ***whole blood****…”* | Please confirm that all your study participants received whole blood and not red cell concentrates or other individual blood products. | Yes we sought out patients who received whole blood and not other blood components |
| 93-94 | *“…study conducted at the Uganda Cancer Institute (UCI) and the internal medicine unit of…”* | It looks like most of your study participants were immunocompromised (HIV and/or malignancy). I would suggest you add a line or so in the background section on the risks of transfusing whole blood in such population. | Thank you. We have revised the paragraph and included “Anaemia and impaired immunity is also associated with decreased survival and progression of the underlying disease…” in lines 72-73 |
| 99-100 | *“…tumours are* ***treated*** *at the UCI; whereas patients with general internal medicine conditions are* ***taken care of*** *in MNRH* | I would suggest you use the word “managed” for both “treated” and “taken care of” for uniformity. | Addressed in lines 104 – 105 |
| 100-101 | *“…generally follow the same guidelines from the Ugandan ministry of health which recommends…”* | Please add a comma after the word “health”. | Addressed, line 106 |
| 103 | *“…haemoglobin level is <7 g/dL or 6 for patients with…”* | 6 what? Please add the units. | Addressed, line 108  6g/dL |
| 104 | *“Blood components for the two hospitals are* ***prepared*** *and provided…”* | Please use the word “processed” instead of “prepared”. | Addressed, line 109 |
| 105-107 | *“Prior to delivery to the hospitals, all blood is serologically tested to be negative for human immunodeficiency virus, hepatitis B and C, and syphilis.”* | Please rephrase:  i.e., "Every donated blood products/ unit is tested for HIV, hepatitis B, hepatitis C, and syphilis, and can only be issued to the hospital once all the tested serological markers are negative".  Please ensure that no nucleic acid testing is performed on these units before committing yourself to only serological testing. | Addressed, lines 110-112  Thank you.  Nucleic acid test has recently been included at the UBTS to improve transfusion safety  Lines 112 -113 |
| 110-111 | *“…immediate spin cross-match – all performed using the tile method which is standard of care in SSA settings”* | Which one is the standard of care? The time method or just the cross-match? | Edited out the “standard of care in SSA settings” to avoid confusion |
| 131 | *“…median and IQR for nonparametric data”* | Please spell IQR in full the first time. | Addressed, line 138 |
| 144-145 | *“Baseline haemoglobin levels, stages of cancers and HIV characteristics were not included since they were not consistently stated…”* | Baseline haemoglobin could have been a very valuable piece of information in this study. I’m not sure how readily available this information can be from the blood bank? | We agree this was a big concern. Unfortunately, it is very challenging to again retrieve the patients’ file to obtain this information. Besides, not all files had a baseline haemoglobin as some patients were transfused on clinical grounds. It’s even harder to get them from the hospital blood banks. |
| 151-152 | *“The top five common diagnoses were isolated HIV related anaemia…”* | Parvovirus B19 infection is very common in this group of patients, and requires treatment with IVI immunoglobulins rather than blood transfusion. Is there any information in this regard? | There was no information on parvovirus B19 |
| 153 | *“…unexplained anaemias, 186 (9.2)…”* | Please add the units for 9.2 | Addressed, line 160 |
| 154 | *“Most patients received 1 unit of blood…”* | Write “1” in words.  Write “whole blood” if it is whole blood. | Addressed, line 161 |
| 209 | *“In this retrospective study of patients transfused with whole blood in Uganda…”* | Again, please confirm that all your study participants received whole blood and not red cell concentrates or other individual blood products. | Affirmative |
| 227 | *“A systematic study to assess the association of* ***red blood cell transfusion and*** *in-hospital mortality…”* | You probably might want to also look at the literature about “whole blood” transfusion. | Sentence added for whole blood on lines 230-231 |
| 231 | *“(red blood cells, RBCs)”* | Please omit “red blood cells” | Addressed, line 233 |
| 250 | *“…all patients in our study received non-leucoreduced whole blood.”* | Please add a line or so in the background about the risks of transfusing “non-leucoreduced whole blood.” | Addressed in lines 74-75 |
| 260-262 | *“…the underlying disease condition in our study may simply reflect the inherent influence of the specific disease entity on mortality,* ***rather than the effect of blood transfusion.”*** | I would suggest that the authors add in the background:   - The risks of blood transfusion. - Advantages and disadvantages of transfusing whole blood vs RBC (leuco-depleted vs unfiltered). - Clinical impact of anaemia in immunocomromised patients   Please add in the discussion whether your hospitals do have any “haemovigilance” committee, or any designated person who oversees the transfusion-associated reactions. | First paragraph of background, lines 68-75 has been revised to capture this concern  Thank you we have included information on haemovigilance under study setting, Line 117. |
| 278-279 | *“…patients who received additional blood had reduced mortality rates.”* | Is it possible that “inadequate” transfusion might have been a contributing factor in the higher mortality amongst those participants who received only one unit of whole blood? | Yes, we have highlighted this in lines 220-221: “…If blood supply was an issue then the mortality rate may suggest inadequate transfusion in patients who might have had severe anaemia…” |

**Reviewer 2**

**Your comments are in normal font while our responses are in italics and preceded by “Response”**

Background:
The paper is on whole blood transfusions yet the background is more focused on blood components transfusion. Appreciating there is not much published on whole blood transfusions, the context in which it is commonly used (e.g. resuscitation and pediatrics) could have been included in the introduction. Any papers that have compared whole blood vs blood component transfusions and their conclusions would have added more weight. The comparing of mortality rate of this paper with other published data on patients who were transfused blood components may be challenged.

***Response:*** *Thank you for this suggestion. Background has been updated to include situation where whole blood is used and a study on whole blood vs red cell concentrates (Lines 76-79). We also accept the limited literature on whole blood transfusion.*

Study design and setting:
Line 95- the inclusion of surgical wards/trauma would have provided a better comparison with published data.

***Response:*** *We acknowledge this as a limitation for our study.*

Eligibility criteria:
Line 115- seasonality in supply and transfusion practices. Elaboration on this would be of value as it is a local experience?

***Response:*** *Thank you, a sentence is added in lines 122-123 stating “Blood collection is usually at its peak during the seasons when student-donors are in school.”*

Data analysis:
Line 144- the exclusion of baseline hemoglobin, although mentioned as a limitation, is a crucial omission. The degree of anemia is known to be an important risk factor for mortality and a marker of underlying disease.

***Response:*** *Thank you, we agree. We included this under discussion lines 227-228 “Anaemia is an independent mortality indicator”*

Mortality rate:
Line 179- the 148 patients who died on the same day of transfusion, further analysis of this group would have been of value and possibly contributed to additional risk factors

***Response:*** *Thank you. Unfortunately, we did not collect further data on this group of patients.*
