# Supplementary material for: Mortality and its associated factors in transfused patients at a tertiary hospital in Uganda
Source: PLoS One. 2022 Sep 22;17(9):e0275126. doi: 10.1371/journal.pone.0275126 (PMC9499229; doi:10.1371/journal.pone.0275126)
Supplement: Attachment — Submitted filename: Response to comments.docx [file pone.0275126.s004.docx]

**RESPONSE TO EDITORIAL COMMENTS**

Dear Sir/Madam,

Thank you for your comments. We have addressed the concerns as follows (your comments are in regular fonts and our responses follow immediately):

We've checked your submission and before we can proceed, we need you to address the following issues:

1. We note that your uploaded Cover Letter is not a Cover Letter but a Response to Reviewers file. It is important that you include a cover letter with your manuscript. Please ensure that this letter is addressed specifically to PLoS ONE. Please also include
* why this manuscript is suitable for publication in PLoS ONE.
* how does your paper provide a worthwhile addition to the scientific literature?
* how does your paper relate to previously published work?
* which types of scientists do you believe will be most interested in your study?

Please also note that the Cover Letter and Response to Reviewers file should be uploaded as two separate files.

**Response(s):** Addressed


2. Thank you for updating your data availability statement. You note that your data are available within the Supporting Information files, but no such files have been included with your submission. At this time we ask that you please upload your minimal data set as a Supporting Information file, or to a public repository such as Figshare or Dryad.
Please also ensure that when you upload your file you include separate captions for your supplementary files at the end of your manuscript.
As soon as you confirm the location of the data underlying your findings, we will be able to proceed with the review of your submission.

**Response(s):** Addressed

Regards,

Clement Okello,

Study PI
